# Developmental Changes in Time and Space Promote Evolutionary Diversification of Flowers: A Case Study in Dipsacoideae

**DOI:** 10.3389/fpls.2017.01665

**Published:** 2017-10-06

**Authors:** Somayeh Naghiloo, Regine Claßen-Bockhoff

**Affiliations:** Instituts für Organismische und Molekulare Evolutionsbiologie, Johannes Gutenberg Universität, Mainz, Germany

**Keywords:** Dipsacoideae, merosity, organ initiation sequence, meristem quantification, spatial constrains, heterochrony, flower diversification

## Abstract

Diversification in flower shape and function is triggered by the high plasticity of flower meristems. Minute changes in space and time can profoundly affect the formation of adult structures. Dipsacoideae provides an excellent model system to investigate the evolutionary aspects of temporal and spatial changes in flower development due to its small size, the resolved phylogenetic framework, and significant diversity of perianth form and merosity. In the present study, we investigated the sequence of floral organ initiation and quantified the interactions between flower meristem expansion and petal primordium size in eight species representing two major clades of Dipsacoideae. Our quantitative study indicates the plasticity of the flower meristem for the regulation of pentamery either due to a decrease in petal primordium size (*Scabiosa*) or an increase in flower meristem size (*Pterocephalus* and *Lomelosia*) compared to tetramerous flowers. According to our results, temporal shifts of organ initiation during flower evolution contribute to the morphological diversity of perianth. Sepal reduction in members of the Dipknautids is paralleled by a delay in sepal initiation. The multiplication of sepals in *Lomelosia* and *Pterocephalus* is correlated with an extension of initiation time. Some heterochronies in early development do not affect adult morphology. The effects of a temporal change in early development can be enhanced, reduced, or eliminated by later changes of the growth rate during development. Our results confirm the hypothesis that the interaction between timing and space plays an important role for evolutionary diversification of flowers.

## Introduction

Understanding the evolution of flower diversity has been a main challenge for evolutionary developmental (evo–devo) biologists over the past 20 years (Becker et al., 2011). Differential activity and high plasticity of flower meristems contribute to the great diversity of flower shapes and functions (Leins and Erbar, 2010; Ronse De Craene, 2010; Claßen-Bockhoff, 2016). The flower meristem produces flower organs over time and through space. The issue of space and time thus profoundly affects the diversification of flowers.

Heterochrony, i.e., changes in the timing and rate of developmental events, provides a plausible explanation for the diversification of characters in the course of flower evolution (Li and Johnston, 2000). Since Haeckel (1875) introduced the concept of ontogenetic plasticity, heterochrony has been extensively studied as a source of animal variation and evolution. Takhtajan (1976) was among the first who introduced the concept in plants. He emphasized the role of “terminal abbreviation of development” (neoteny in terms of Takhtajan) in angiosperm evolution. The heterochronic changes have been the subject of several plant evolutionary studies through the twentieth century (reviewed in Li and Johnston, 2000). Most previous cases of heterochrony have been documented through the quantitative analysis of organ shape and size during flower development (Li and Johnston, 2000) which fall into the definition of “growth heterochrony” (Smith, 2001). However, the interspecific comparison of sequence of developmental events or “sequence heterochrony” can provide more convincing support for heterochronic changes (Smith, 2001). The comparative developmental studies of flowers within a robust phylogenetic framework can reveal changes in the start or end point, growth rate, or sequence of early developmental events which promote the morphological diversity of flowers at maturity.

Change in the spatial conditions of a meristem is another fundamental process for flower diversification. Flower meristems differ from shoot apical meristems (SAMs) in their growth capacity. While SAMs have a central zone with initial (or stem) cells, this zone is lacking in flower meristems. Flower meristems thus lack the apical growth of SAMs, but instead extend by an overall internal cell division activity (Claßen-Bockhoff and Bull-Hereñu, 2013; Claßen-Bockhoff, 2016). Given that meristem expansion occurs parallel to the initiation of organ primordia, it changes the density of already initiated primordia and provides space for additional primordia (Szpak and Zagórska-Marek, 2011). According to the time and degree of meristem expansion and the size of organ primordia, a change in merosity (i.e., the number of organs per whorl) or formation of new organs can be induced (Claßen-Bockhoff and Meyer, 2016; Naghiloo and Claßen-Bockhoff, 2016; Ronse De Craene, 2016).

**FIGURE 1:**
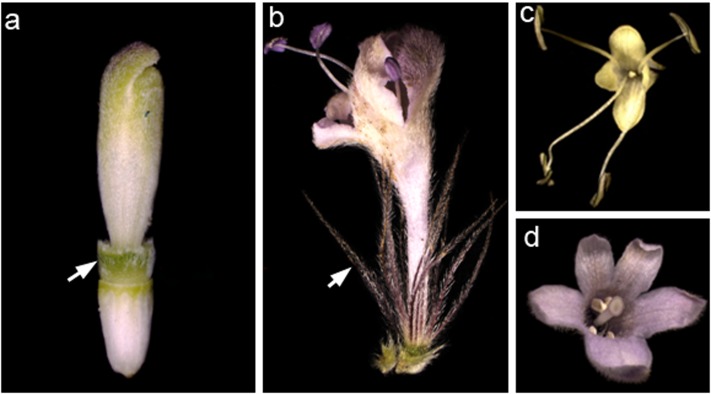
Variation of perianth morphology in Dipsacoideae. **(a,b)** Cup-shaped (*Dipsacus*) versus bristle-like (*Pterocephalus*) sepals (arrows). **(c,d)** Tetramerous (*Cephalaria*) versus pentamerous (*Pterocephalus*) corolla.

Dipsacoideae is a small group (14 genera) in the Valerina clade of Caprifoliaceae (Donoghue et al., 2001). Given the small size of the group, the resolved phylogenetic framework, and the significant diversity of perianth form and merosity, the subfamily provides an excellent model system to investigate the issue of time and space in flower evolution. Based on molecular phylogenetic studies, Dipsacoideae split into two clades, the small *Bassecoia* clade and its large sister clade comprising the Dipknautids and Scabioseae as sister groups. The flowers are characterized by the formation of an epicalyx as a “key innovation” that spurred the adaptive radiation of Dipsacoideae in the Mediterranean Basin (Verlaque, 1984). The morphology of the epicalyx and calyx has undergone several modifications within the Dipsacoideae in response to different seed dispersal syndromes. Most species in Scabioseae have elaborated epicalyx with “wing-like” appendages and tend to modify sepals as bristles (**Figure 1b**). This combination of characters facilitates the wind dispersal (Mayer, 1995; Caputo et al., 2004). However, in most members of Dipknautids clade, the epicalyx is simple and the calyx is reduced to a rim indicating that these structures are not directly involved in dispersal (**Figure 1a**). Instead, the majority of species rely on a combination of rigid, often acuminate receptacular bracts in the capitulum, and of elastic stems which allow short-distance projection of seeds (Caputo et al., 2004). Along with the diversification of the calyx, the merosity of the corolla tends to vary within Dipsacoideae (**Figure 1**). While most Scabioseae are characterized by pentamerous corollas (**Figure 1d**), tetramerous corollas (**Figure 1c**) are often found in *Bassecoia* and Dipknautids (Carlson et al., 2009).

In the present study, we investigated the sequence of floral organ initiation and the interaction between flower meristem expansion and petal primordia size in eight species representing two major clades of Dipsacoideae. We aim to test the hypothesis that (i) temporal shifts in the development of flowers (heterochrony) contribute to perianth diversification in the course of evolution, and that (ii) spatial conditions are involved in the genesis of different merosities.

## Materials and Methods

### Scanning Electron Microscopy

Buds of inflorescences and flowers of the following species were collected in May–August 2015 at the Botanical Garden of the Johannes Gutenberg University Mainz (Germany): *Succisa pratensis* Moench., *Succisella inflexa* (Kluk) Beck., *Cephalaria transsylvanica* (L.) Schrad., *Dipsacus fullonum* L. and *Knautia arvensis* (L.) Coult. (all Dipknautids clade), *Lomelosia palaestina* (L.) Raf., *Pterocephalus papposus* (L.) Coult., and *Scabiosa ochroleuca* L. (all Scabioseae clade).

Plant material was stored in 70% EtOH and dehydrated in an ascending alcohol–acetone series. The samples were critically point-dried (BAL-TEC CPD030), sputter-coated with gold (BAL-TEC SCD005), and observed under the scanning electron microscope (ESEM XL-30 Philips). All steps were conducted according to the manufacturer’s protocols.

### Morphometric Analysis

**FIGURE 2:**
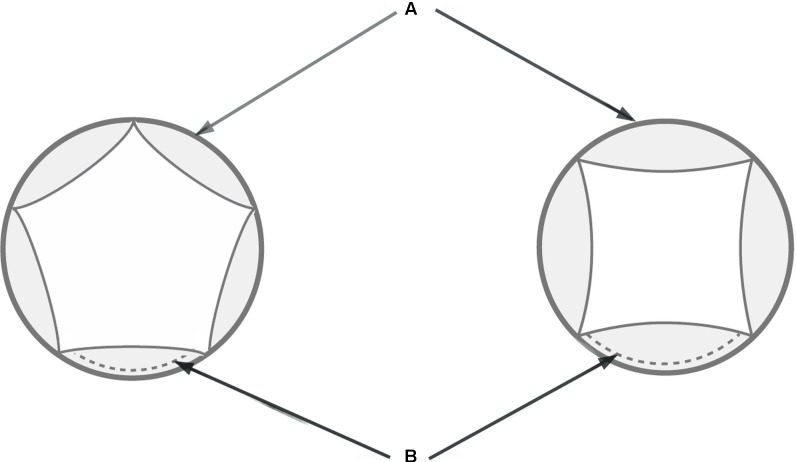
Schematic diagram of tetramerous and pentamerous flowers showing the measured traits. **(A)** Extent of a flower meristem soon after petal initiation. **(B)** Length of petal’s abaxial extent.

As most studied species have dimorphic flowers in the center versus periphery of the head, we only used samples from central flowers for the interspecific comparison. In order to calibrate the measurements, we selected the earliest stage where all petals are visible (**Figure 2**). The following measurements were conducted in tetramerous and pentamerous species based on SEM micrographs: (i) extent of flower meristem (**Figure 2A**) and (ii) length of petal’s abaxial extent (**Figure 2B**). All photos were taken in top view in a standard manner. Petal length is presented as an average of all petals per flower. The measurements were carried out in five to seven flowers per species. We used Digimizer software^1^ (MedCalc Software bvba, Belgium) for measurement and SPSS statistic 22v for statistical analyses. A single-factor analysis of variance (ANOVA) was used to test for significant differences of traits between species. This was followed by Duncan’s test for multiple comparisons of means between species. Results were considered significant for *p* < 0.05.

## Results

Results indicate that the diversity in calyx morphology and flower merosity is related to the sequence of organ initiation and flower meristem size. This is evident from ontogenetic and morphometric studies.

### Ontogenetic Studies

All species share a set of ontogenetic characters, but differ in species-specific ones.

#### Common Pattern of Flower Development

**FIGURE 3:**
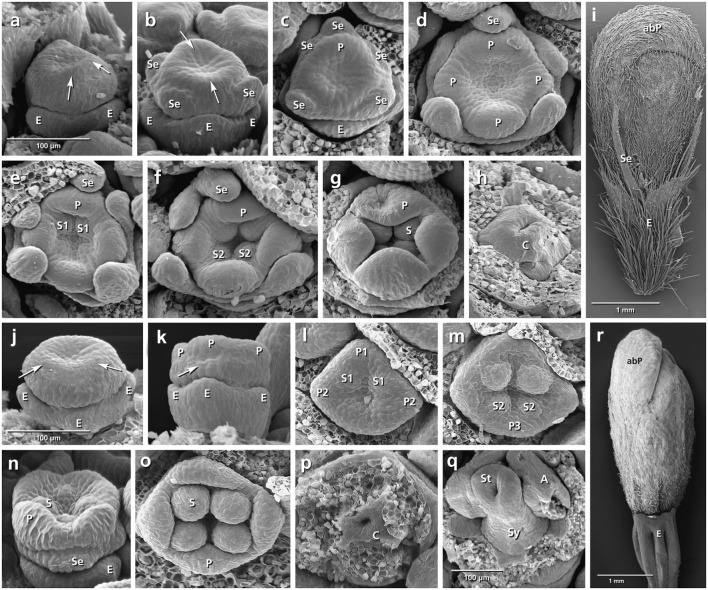
Flower development in *Succisa pratensis* **(a–i)** and *Succisella inflexa* **(j–r)**. Adaxial side is toward the top in **(a–h,i,m,o)**. **(a,b)** Initiation of epicalyx, appearance of sepals, and corolla ring (arrows). **(c–f)** Initiation of petals and stamens. **(g,h)** Enlargement of petals and appearance of carpel ring meristem. **(i)** Bristle-like sepals in mature flower. **(j–r)** The same developmental stages in *S. inflexa*. Note the late initiation of the calyx ring in **(k)** (arrow), the overlapping petal–stamen whorls in **(l**,**m)**, and the inconspicuous sepals in **(r)**. Abbreviations: abP, abaxial petal; C, carpel; E, epicalyx; O, ovary; P, petal; S, stamen; Se, sepal; St, stigma; Sy, style. Same scale for **(a–h)** and **(j–p)**.

**FIGURE 4:**
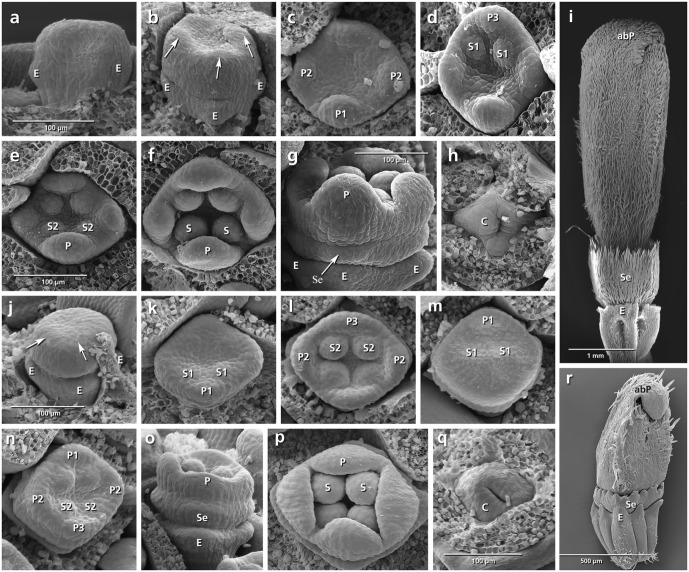
Flower development in *Dipsacus fullonum* **(a–i)** and *Cephalaria transsylvanica* **(j–r)**. Adaxial side is toward the top in **(c–f,k–n)**. **(a,b)** Appearance of epicalyx and corolla ring (arrows). **(c–f)** Initiation of petals and stamens. **(g)** Formation of calyx ring (arrow) higher than epicalyx. **(h)** Appearance of carpel ring meristem. **(i)** Development of calyx as a cup-shaped hairy structure in mature flower. **(j–r)** The same developmental stages in *C. transsylvanica*. Note the overlapping petal stamen whorls in **(m,n)**, and toothed cup-shaped calyx in **(r)**. Abbreviations: abP, abaxial petal; C, carpel; E, epicalyx; O, ovary; P, petal; S, stamen; Se, sepal. Same scale for **(a–d)**, **(e,f)**, **(g,h)**, and **(j–p)**.

**FIGURE 5:**
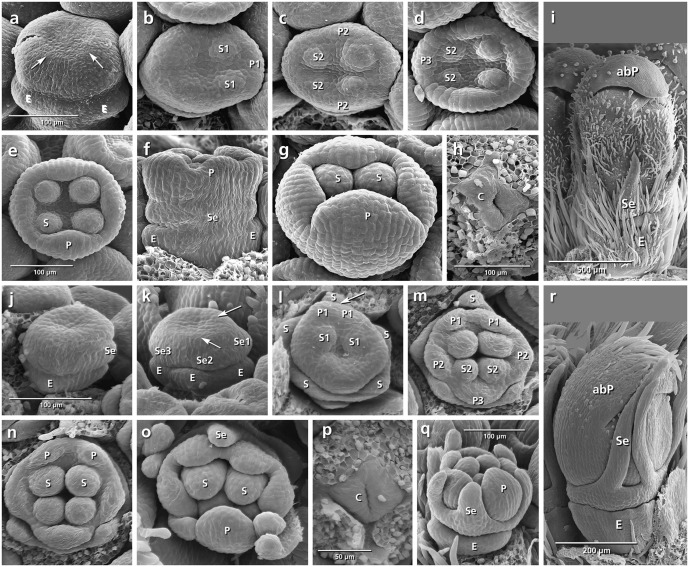
Flower development in *Knautia arvensis* **(a–i)** and *Scabiosa ochroleuca* **(j–r)**. Adaxial side is toward the top in **(b–e,l–o)**. **(a)** Appearance of epicalyx and corolla ring (arrows). **(b–e)** Initiation of petals and stamens. **(f)** Formation of calyx ring higher than epicalyx. **(g)** Enlargement of petals. **(h)** Appearance of carpel ring meristem. **(i)** Development of calyx as small hairy bristles. **(j–r)** The same developmental stages in *S. ochroleuca*. Not the early initiation of sepals in **(j,k)**, and development of enlarged bristles in **(r)**. Abbreviations: abP, abaxial petal; C, carpel; E, epicalyx; O, ovary; P, petal; S, stamen; Se, sepal. Same scale for **(a–d)**, **(e–g)**, and **(j–o)**.

**FIGURE 6:**
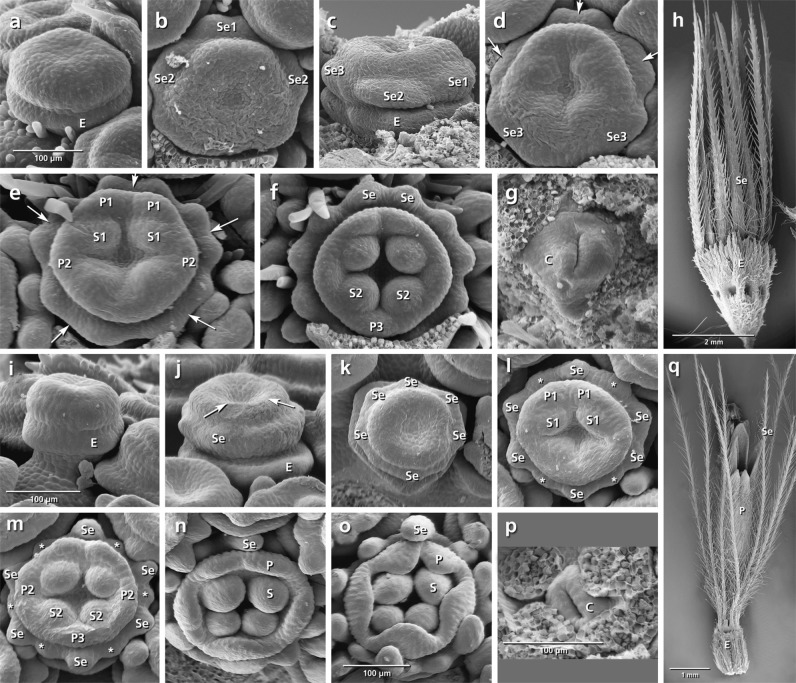
Flower development in *Lomelosia palaestina* **(a–h)** and *Pterocephalus papposus* **(i–q)**. Adaxial side is toward the top in **(b)**, **(d–f)**, and **(k–o)**. **(a)** Appearance of epicalyx. **(b)** Initiation of primary sepal primordia starting from the adaxial side. **(c–f)** Subdivision of primary sepal primordia (arrows) and initiation of petals and stamens starting from the adaxial side. **(g)** Appearance of carpel ring meristem. **(h)** Development of hairy bristle-like sepals covering the corolla. **(i–q)** The same developmental stages in *P. papposus*. Note the initiation of six primary sepal lobes and later formation of new sepal lobes (asterisks) between the primary lobes in **(k–m)**. Abbreviations: abP, abaxial petal; C, carpel; E, epicalyx; O, ovary; P, petal; S, stamen; Se, sepal. Same scale for **(a–g)** and **(i–n)**.

The epicalyx elements are always the first organs initiated (**Figures 3a,j**, **4a,j**, **5a,j**, **6a,i**: E). Calyx initiation differs as specified below (see section “Species-Specific Deviations from the Common Pattern of Development”). The corolla appears as a ring-like zone (**Figures 3a,b,j**, **4b,j**, **5a,j**, **6a,i**: arrows), soon followed by the initiation of four or five corolla lobes (**Figures 3d,m**, **4c,l**, **5d,m**, **6e,m**: P). Initiation of petal and stamen primordia proceeds simultaneously in a unidirectional order, starting from the adaxial side and continuing toward the abaxial side (**Figures 3e,f,l,m**, **4d,e,m,n**, **5l,m**, **6e,f,l,m**: P, S). While the petals and stamens enlarge, the carpel ring is initiated and starts to form the inferior ovary (**Figures 3h,p**, **4h,q**, **5h,p**, **6g**, p: C).

#### Species-Specific Deviations from the Common Pattern of Development

Variation in both the timing and sequence of organ initiation is found in the studied species. This is particularly remarkable during calyx development.

##### Dipknautids clade

In all studied members of Dipknautids, the calyx initiates as a ring meristem. Its formation is delayed compared to petal and stamen primordia. *Succisa* is an exception in which the sepals appear as five individual lobes before the appearance of petal and stamen primordia. All members of the clade share the tetramerous corolla.

###### Succisa pratensis (**Figures 3a–i**)

The initiation of the sepals precedes the formation of the inner organs. The first three sepal lobes appear simultaneously on the abaxial and adaxial side of the flower meristem (**Figure 3b**: Se) followed by the formation of the two lateral sepal lobes (**Figure 3c**: Se). No overlap in the time of initiation is observed between petal and stamen primordia (**Figure 3d**) resulting in a centripetal order of initiation. In mature flowers, calyx lobes appear as small bristles at the base of corolla (**Figure 3i**).

###### Succisella inflexa (**Figures 3j–r**)

The calyx appears as a ring-like structure on the top of the epicalyx, simultaneously with the formation of petal and stamen primordia (**Figure 3k**: arrows). Soon after, four inconspicuous calyx lobes develop from the ring meristem (**Figure 3n**: Se). They remain inconspicuous throughout development (**Figure 3r**).

###### Dipsacus fullonum (**Figures 4a–i**)

No overlap in the time of initiation is detected between the petal and stamen whorls (**Figure 4c**). Although the space of the calyx is already provided by meristem extension between the epicalyx and the corolla, the first primordia can be recognized only after the initiation of petal and stamen primordia (**Figure 4g**: arrows). The calyx remains undivided during development and develops as a cup-shaped hairy structure at the base of the corolla (**Figure 4i**: Se).

###### Cephalaria transsylvanica (**Figures 4j–r**)

The petal and stamen whorls appear simultaneously, starting from the abaxial (**Figures 4k,l**: P, S) or adaxial side (**Figures 4m,n**: P, S). The calyx ring then appears between epicalyx and corolla (**Figure 4o**: Se). The calyx develops as a cup-shaped structure with tiny lobes on the top (**Figure 4r**: Se).

###### Knautia arvensis (**Figures 5a–i**)

The order of stamen and petal initiation in *Knautia* differs from the other studied Dipsacoideae. The first petal lobe appears at the lateral side along with the two neighbor alternipetalous stamens (**Figure 5b**: P1, S1). The abaxial and adaxial petals appear later (**Figure 5c**: P2) followed by the formation of the last petal and its neighbor alternipetalous stamens in lateral position (**Figures 5d,e**: P3, S2). Meanwhile, the calyx ring meristem appears between the epicalyx and corolla (**Figure 5f**: Se). With further growth, it differentiates into small bristle-like hairy sepals (**Figure 5i**: Se).

##### Scabioseae clade

The initiation of calyx precedes the formation of inner organs in studied members of Scabioseae. The mature flowers are characterized by their bristle like sepals and pentamerous corollas.

###### Scabiosa ochroleuca (**Figures 5j–r**)

Simultaneously with the formation of the epicalyx lobes, the first sepal primordium appears at the adaxial side (**Figure 5j**: Se1) followed by the lateral and abaxial ones (**Figures 5k,l**: Se2, Se3). At the same time, the first petal primordium appears as a broad meristem at the adaxial side (**Figure 5l**: P?) which is subdivided later into two petal primordia resulting in a pentamerous corolla (**Figures 5l,m**: arrow, P1). Bristle-like sepals enlarge quickly and cover the petals throughout development (**Figure 5r**: Se).

###### Lomelosia palaestina (**Figures 6a–h**)

Concurrently with the formation of epicalyx lobes, five sepal primordia appear unidirectionally from the abaxial side (**Figures 6b,c**: Se, E). Each of them subdivides (**Figures 6d,e**: arrows) to form two separate primordia, resulting in the formation of 10 calyx lobes (**Figures 6e,f**: Se). Bristle-like sepals enlarge quickly and cover the petals throughout development (**Figure 6h**: Se).

###### Pterocephalus papposus (**Figures 6i–q**)

After formation of epicalyx lobes, the calyx appears as a ring meristem on which six sepal lobes differentiate (**Figures 6j,k**: Se). With further growth, new sepal lobes are formed between them which result in the formation of a 12-lobed calyx (**Figures 6l,m**: asterisks).

### Morphometric Analysis (**Figure 7**)

**FIGURE 7:**
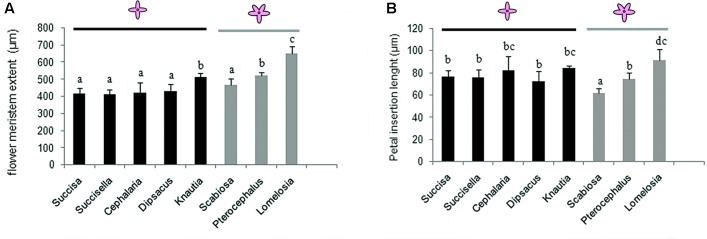
Flower meristem extent **(A)** and petal primordia extent **(B)** in tetramerous flowers of Dipknautids and pentamerous flowers of Scabioseae. Bars represent the mean (*n* = 5–7) + SD. Characters that share the same letter do not differ significantly on the basis of Duncan’s test (*p* < 0.05).

Flower meristem extent shows no significant difference within tetramerous species except for *Knautia* which produces significantly larger flowers. The flower meristem extension varies in the pentamerous species. In *Scabiosa*, it shows no significant difference with the tetramerous flowers, while it is significantly larger in *Pterocephalus* and *Lomelosia*. Within pentamerous flowers, the flower meristem is significantly larger in *Lomelosia* than *Pterocephalus* (**Figure 7A**).

There is no significant difference in the petal insertion length among the tetramerous species. The comparison of petal insertion length in pentamerous species with tetramerous ones shows a significantly smaller size in *Scabiosa*, a significantly larger size in *Lomelosia*, and no significant differences in *Pterocephalus* (**Figure 7B**).

## Discussion

Our study indicates that both spatial and temporal changes are involved in flower evolution in Dipsacoideae.

### Spatial Constrains and Merosity

The question whether tetramery or pentamery is an ancestral condition in Dipsacoideae is still not solved. However, the occurrence of tetramery in basal *Bassecoia* may indicate tetramery as an ancestral condition. This assumption is further enhanced by the study of vascular strands. While only four vascular strands are found in tetramerous flowers (Van Tieghem, 1909; Szabó, 1923), in pentamerous flowers the strand of the fifth lobe results from a deep bipartition of another strand (Molliard, 1895; Alvarado, 1927).

**FIGURE 8:**
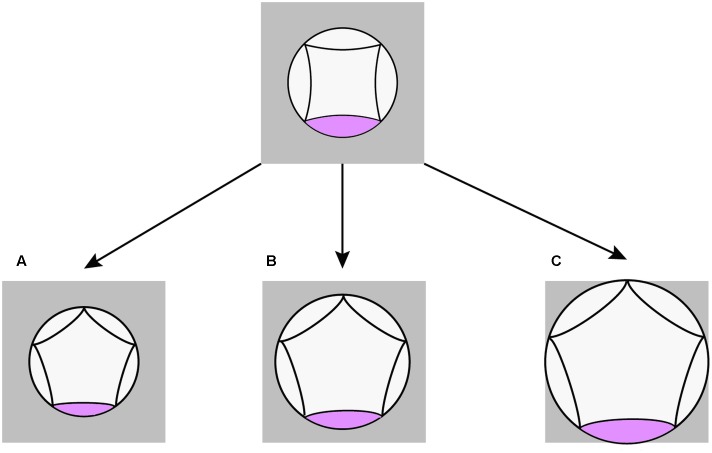
Schematic diagrams showing different spatial changes during evolution of pentamerous flowers in Scabioseae. **(A)** Decrease in petal primordium size in *Scabiosa*. **(B)** Increase in meristem size in *Pterocephalus*. **(C)** Increase in meristem size as well as petal primordium size in *Lomelosia*.

According to our quantitative study, pentamery is achieved by changes in the proportions of the meristem relative to its primordia. The result confirms the importance of the interaction between meristem expansion and primordia formation for the genesis of merosity (Naghiloo and Claßen-Bockhoff, 2016; Ronse De Craene, 2016). Interestingly, such interaction is coordinated in different ways in the pentamerous flowers of Scabioseae (**Figure 8**). In *Scabiosa*, a decrease in the size of petal primordia without any change in meristem size provides space for the formation of a new primordium (**Figure 8A**). The opposite is true for *Pterocephalus* where the required space is provided by an extension of floral meristem without any change in the size of petal primordium (**Figure 8B**). The meristem size also increases in *Knautia* compared to other tetramerous flowers. However, this increase is not enough for the formation of a new primordium indicating that a certain threshold is needed. In contrast to *Scabiosa*, the primordium size increases in L*omelosia* compared to tetramerous flowers (**Figure 8C**). However, the degree of meristem expansion is high enough to offer space for a fifth primordium in *Lomelosia*.

The plasticity of the flower meristem, shown in pentamerous flowers, presents an important evolutionary advantage. It enables the flower meristem to regulate spatial conditions according to the developmental constraints (i.e., the initial size of meristem, the size of outer organs, and the rate of meristem expansion) and changing environment (i.e., light intensity, day length, and humidity).

### Heterochronic Changes and Flower Diversification

**FIGURE 9:**
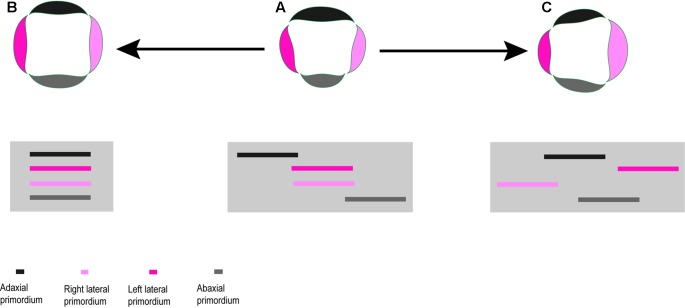
Heterochronic shifts in the order of organ initiation and change in symmetry. **(A)** A zygomorphic flower with sequential initiation of organs. **(B)** Deceleration of organ initiation within whorl results in actinomorphic symmetry. **(C)** Shift in sequence of initiation lead to right–left zygomorphy.

**FIGURE 10:**
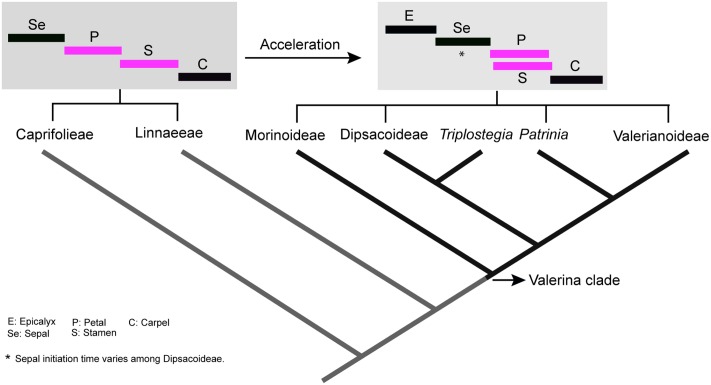
The evolutionary acceleration of flower development in Valerina clade and overlapping initiation of petal–stamen whorls (in pink). Tree based on phylogenetic work of Donoghue et al. (2001). The timing diagrams are based on sequence of flower organs initiation. However, sepal initiation time varies among Dipsacoideae.

**FIGURE 11:**
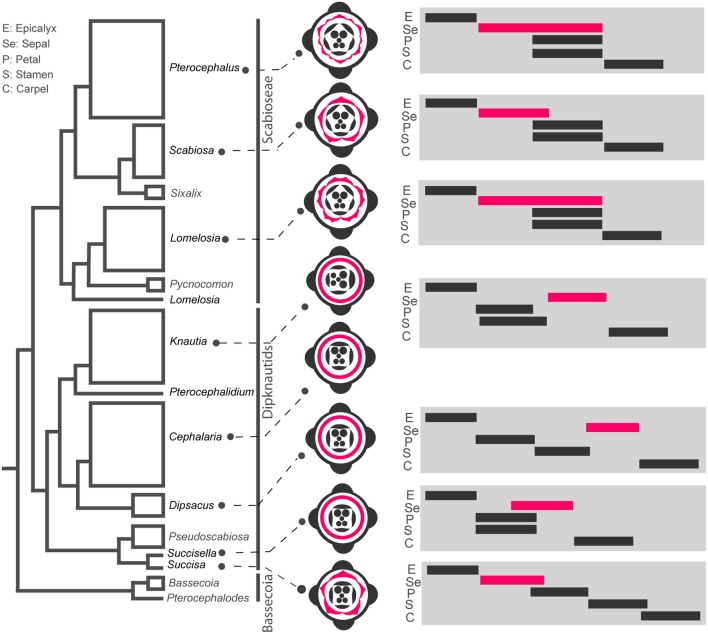
Heterochronic shifts of flower whorls initiation during evolution of Dipsacoideae. The flower diagrams are based on ontogenetic characters and the timing diagrams of flower whorls initiation are shown on the phylogenetic tree (based on Carlson et al., 2009). Note the correlation between reduction of sepal lobes in Dipknautids and delay in sepal initiation, and between multiplication of sepal lobes and extended period of initiation in *Lomelosia* and *Pterocephalus*.

Our interspecific comparison of organ initiation sequence within and between flower whorls revealed several cases of heterochronic shifts during the evolution of flowers on the phylogenetic tree of Dipsacoideae (**Figures 9**–**11**).

#### Shift in Order of Organ Initiation and Change in Symmetry Plan

In most studied taxa, organ initiation in each flower whorl follows a unidirectional order beginning on the adaxial or rarely abaxial side. The sequential initiation of organs is followed by their sequential growth resulting in an abaxial–adaxial size gradient at anthesis. The unidirectional organogenesis is also found in members of the basal Caprifoliaceae (Caprifolieae and Linnaeeae, Roels and Smets, 1996; Landrein and Prenner, 2013) and has been reported as the first expression of zygomorphy in several plant families (Endress, 2012).

The heterochronic shift in the order of organ initiation within a whorl can influence the symmetry pattern. The synchronization of organ initiation results in actinomorphy (**Figure 9B**), while a delayed formation of the left/right side primordia can produce transversal zygomorphy or asymmetry (**Figure 9A**). The latter case is found in *Knautia* which is unique regarding the order of organ initiation among the studied Dipsacoideae. The precocious initiation of the left lateral petal primordium paralleled by the delayed formation of the right lateral petal primordium results in a transversal zygomorphy in *Knautia* (**Figure 9C**). However, this special symmetry disappears during development due to differential organ growth and is replaced by a dorsiventral symmetry.

The reason for the unique initiation sequence in *Knautia* is still wanting but the suppression of bracts may play a role. The possible function of bracts and bracteoles in mediating the onset of the spiral of outer organs has been reported in several developmental studies (Endress, 1994; Haston and Ronse De Craene, 2007; Chandler and Werr, 2014). Among the studied Dipsacoideae, the bracts are only lacking in *Knautia* and *Pterocephalus*. It can be hypothesized that the reduction of bracts in *Knautia* has influenced the order of the first initiated organs, i.e., the petal and stamen primordia. In *Pterocephalus*, which has sepals as the first initiated organs, the lack of bract is paralleled by the formation of a sixth sepal in abaxial position where the bract would normally appear.

#### Shift in Order of Whorls Initiation

The comparison of whorl initiation sequence revealed three types of heterochronic shifts during flower evolution (**Figures 9**, **10**).

##### Synchronization of whorl initiation and acceleration of development

In all genera examined, a high degree of temporal overlap was found between petal and stamen whorl initiation except for *Dipsacus* and *Succisa*. Indeed, all members of the Valerina clade appear to be characterized by a high overlap between petal and stamen initiation (Hofmann and Göttmann, 1990; Roels and Smets, 1996). On the other hand, in the basal clades Caprifolieae and Linnaeeae, all organ whorls appear sequentially and without any overlap in the time of initiation (Roels and Smets, 1996; Landrein and Prenner, 2013). Accordingly, there is an obvious developmental acceleration during the evolution of Caprifoliaceae, resulting in the synchronization of petal and stamen whorls as a specialized trait in the Valerina clade (**Figure 10**). The successive initiation of whorls in *Dipsacus* and *Succisa* may indicate that heterochronic changes are reversible. Alternatively, it is also plausible that acceleration has evolved several times in parallel.

Overlap in the time of organ whorl initiation is reported in several plant families (e.g., Legumes, Tucker, 2003; Naghiloo et al., 2014a,b; Apiaceae, Ajani et al., 2016). It often characterizes flowers with sequential initiation of organs within whorls. Hypothetically, such an overlap is a mechanism through which the deceleration of organ initiation within whorls is compensated by the acceleration of whorls initiation.

##### Shift in the sequence of whorl initiation and organ reduction

In *Succisa*, five individual sepal lobes appear which is comparable with basal subfamilies of Caprifoliaceae (Caprifolieae and Linnaeeae, Roels and Smets, 1996; Landrein and Prenner, 2013). While the formation of sepal lobes is suppressed in other members of the Dipknautids, well-developed sepal lobes are found in the Scabioseae. Considering the evolution of the calyx based on the phylogenetic tree of Dipsacoideae, we can see a clear tendency of differentiation in the sepals of the Scabioseae (**Figure 11**). Sepal reduction in members of the Dipknautids is paralleled by a delay in sepal initiation. As is shown in timing diagrams (**Figure 11**), the initiation of the calyx primordium precedes the formation of petal and stamen primordia in the Scabioseae clade and *Succisa*, whereas in other members of the Dipknautids the calyx primordium initiates after or simultaneously with the formation of petal and stamen primordia (**Figure 11**). The delayed initiation of the calyx primordium may influence the availability of space due to the presence of inner organs, and as a consequence the formation of sepal lobes is suppressed. Developmental studies in Asteraceae (Dadpour et al., 2012), Apiaceae (Ajani et al., 2016), and Rubiaceae (Naghiloo and Claßen-Bockhoff, 2016) are in agreement with this hypothesis.

##### Prolongation of whorl initiation time and increase in organ number

While *Scabiosa* is characterized by a pentamerous calyx, the number of calyx lobes increases to 10 in *Lomelosia* and 12 in *Pterocephalus*. As is shown in the timing diagrams (**Figure 11**), the multiplication of sepals in *Lomelosia* and *Pterocephalus* is correlated with the prolonged initiation time of sepals compared to *Scabiosa* (**Figure 11**). This extension is achieved by the delay in the offset of sepal initiation, resulting in a partial time overlap of sepals and inner whorls. Interestingly, *Lomelosia* and *Pterocephalus* use different mechanisms for sepal multiplication which is influenced by the time of meristem expansion. The considerable expansion of the meristem simultaneously with the initiation of primordia in *Lomelosia* results in primordia enlargement and their later subdivision, while later expansion of meristem in the area between already initiated primordia in *Pterocephalus* produces space for the formation of new primordia. Such a delay in the expansion of meristem in *Pterocephalus* compared to *Lomelosia* can also be considered as a heterochronic event.

Summarizing, our study indicates that the shift in developmental timing occurs frequently during flower evolution. It provides an explanation for most of morphological diversity namely symmetry, reduction of organs, or increase in organ number. However, some of the heterochronies do not appear to affect adult morphology like the shift in the sequence of organ initiation in *Knautia*. This may be interpreted as the expression of changing growth rate during development. The effects of a timing change in early development can be enhanced, reduced, or eliminated by later changes of growth rate during development. The interaction between timing and rate is thus important to morphogenesis and should be considered for the interpretation of the heterochronic events.

Understanding the underlying mechanism of heterochrony would be a main challenge for future studies. According to recent studies, the sequential and antagonistic changes in the level of two microRNAs (miR156 and miR172) during development act as important signals for timing of developmental events (Chuck et al., 2007; Wu et al., 2009). Interestingly, the level of these microRNAs is known to be controlled by environmental stimuli factors (Geuten and Coenen, 2013). These novel findings are important steps toward understanding the origin of heterochronic evolution in plants. Dipsacoideae presents an appropriate model experimental system to study the molecular basis of heterochronic changes during flower evolution.

## Conclusion

Our study confirms the hypothesis that heterochronic changes like acceleration, deceleration, prolongation, and shift in the sequence of organ initiation contributed to flower diversification during evolution. Indeed, the heterochronic events establish the duration of meristem competency for organ initiation, while the spatial constrains and relations between meristem expansion and primordia initiation determine where and how new organs will appear. The final morphology of flowers is thus indicated by the interaction between timing and space.

## Author Contributions

SN and RC-B have substantial contributions to the conception or design of the work, drafting the manuscript, or critically revising it.

## Conflict of Interest Statement

The authors declare that the research was conducted in the absence of any commercial or financial relationships that could be construed as a potential conflict of interest.

## Footnotes

1 http://www.download.hr/download-digimizer.html
